# The NA_v_1.7 blocker protoxin II reduces burn injury-induced spinal nociceptive processing

**DOI:** 10.1007/s00109-017-1599-0

**Published:** 2017-10-23

**Authors:** Jose Vicente Torres-Pérez, Pavel Adamek, Jiri Palecek, Marcela Vizcaychipi, Istvan Nagy, Angelika Varga

**Affiliations:** 1 Nociception Group, Section of Anaesthetics, Pain Medicine and Intensive Care, Department of Surgery and Cancer, Imperial College London, Chelsea and Westminster Hospital, 369 Fulham Road, London, SW10 9NH UK; 2 Department of Functional Morphology, Institute of Physiology, The Czech Academy of Sciences, Prague, Czech Republic; 3 Department of Physiology, Faculty of Science, Charles University, Prague, Czech Republic; 4 Department of Anaesthetics, Chelsea and Westminster Hospital NHS Trust, 369 Fulham Road, London, SW10 9NH UK; 5 MTA-DE-NAP B-Pain Control Research Group, Department of Anatomy, Histology and Embryology, University of Debrecen, Debrecen, 4012 Hungary

**Keywords:** Pain, p-ERK1/2, Primary sensory neuron, p-S10H3, Spinal cord

## Abstract

**Abstract:**

Controlling pain in burn-injured patients poses a major clinical challenge. Recent findings suggest that reducing the activity of the voltage-gated sodium channel Na_v_1.7 in primary sensory neurons could provide improved pain control in burn-injured patients. Here, we report that partial thickness scalding-type burn injury on the rat paw upregulates Na_v_1.7 expression in primary sensory neurons 3 h following injury. The injury also induces upregulation in phosphorylated cyclic adenosine monophosphate response element-binding protein (p-CREB), a marker for nociceptive activation in primary sensory neurons. The upregulation in p-CREB occurs mainly in Na_v_1.7-immunopositive neurons and exhibits a peak at 5 min and, following a decline at 30 min, a gradual increase from 1 h post-injury. The Na_v_1.7 blocker protoxin II (ProTxII) or morphine injected intraperitoneally 15 min before or after the injury significantly reduces burn injury-induced spinal upregulation in phosphorylated serine 10 in histone H3 and phosphorylated extracellular signal-regulated kinase 1/2, which are both markers for spinal nociceptive processing. Further, ProTxII significantly reduces the frequency of spontaneous excitatory post-synaptic currents in spinal dorsal horn neurons following burn injury. Together, these findings indicate that using Na_v_1.7 blockers should be considered to control pain in burn injury.

**Key messages:**

• Burn injury upregulates Na_v_1.7 expression in primary sensory neurons.

• Burn injury results in increased activity of Na_v_1.7-expressing primary sensory neurons.

• Inhibiting Na_v_1.7 by protoxin II reduces spinal nociceptive processing.

• Na_v_1.7 represents a potential target to reduce pain in burn injury.

**Electronic supplementary material:**

The online version of this article (10.1007/s00109-017-1599-0) contains supplementary material, which is available to authorized users.

Burn injury is associated with moderate to severe pain that represents a significant clinical challenge [1]. The lack of effective pain management in burn-injured patients can lead to long-term consequences including the development of anxiety, depression, post-traumatic stress disorder and chronic pain [1]. Therefore, there is a need for the development of novel analgesic approaches to control pain in burn-injured patients.

A series of mediators produced and released during inflammation that ensues after burn injury activate a major sub-set of primary sensory neurons [1, 2]. The resulting generation and propagation of action potentials initiate nociceptive processing in the central nervous system and lead to the experience of pain. Voltage-gated Na^+^ channels (Na_v_), distinguished by their alpha sub-units [3–5], are pivotal for the generation and propagation of action potentials in neurons [6]. Recent studies identified Na_v_1.7 as a putative key molecule for the development of heat hypersensitivity in burn injury [4, 7, 8]. Therefore, in the present work, we examined whether specific blockade of Na_v_1.7 with the selective toxin, protoxin II (ProTxII) [9, 10], is a feasible target to reduce nociceptive processing in burn injury.

## Materials and methods

### Animals, burn injury and treatment

We obeyed the UK Animals (Scientific Procedures) Act 1986, the guidelines of the revised National Institutes of Health *Guide for the Care and Use of Laboratory Animals*, Directive 2010/63/EU of the European Parliament and of the Council on the Protection of Animals Used for Scientific Purposes and the Committee for Research and Ethical Issues of IASP published in Pain, 16 (1983) 109–110 and adhered to Good Laboratory Practice and ARRIVE guidelines. Procedures were approved by veterinary services at all relevant institutions. Every effort was made to minimise the number of animals used and the potential distress. Animals were housed in climate-controlled rooms, on a 12 h light/dark cycle, with food and water ad libitum. In total, 36 male Sprague-Dawley rats (150-200 g), 5 male Wistar rats (P21), 2 wild-type (WT, ~ 22 g) mice and 2 mice lacking Na_v_1.7 in Nav1.8-expressing cells (Nav1.7cKO, ~ 22 g; [5]; both types on C57BL/6 background) were used.

Burn or sham injury was induced as described previously [11, 12]. Briefly, animals were anaesthetised by intraperitoneal urethane (0.02 mg/g) or isoflurane (3%, for spinal cord slice preparation) and one of the hind paws (both paws for spinal cord slices) was immersed into 60 or 37 °C water up to the knee for 2 min. Anaesthesia was maintained for up to 180 min post-injury. Following intraperitoneal sodium pentobarbital, animals were either transcardially perfused with saline then 4% paraformaldehyde or the L4–L5 dorsal root ganglia (DRGs) were dissected from both the ipsilateral and contralateral sides. ProTxII (0.1 mg/kg; Tocris) or morphine (3 mg/kg; Sigma) was injected intraperitoneally 15 min before or after the injury.

### Western blotting

The protocol included tissue homogenisation with a pestle and mortar in ice-cold RIPA buffer (Amresco) and protease inhibitor cocktail (Sigma-Aldrich), sonication for 1 h at 4 °C, spinning for 30 min at 14,000 rpm at 4 °C and denaturing at 95 °C for 5 min. NuPAGE Novex 4–12% Bis-Tris protein gels (Invitrogen, UK) were used for separation. After transfer to PVDF membranes (Invitrogen, UK), samples were incubated in 5% non-fat milk powder (Sigma, UK) for 1 h at room temperature, then in anti-Na_v_1.7 and anti-β-tubulin III antibodies at 4 °C overnight followed by incubation in secondary antibodies at room temperature for 1 h and visualisation with the Luminol kit (Santa Cruz, USA; Supplementary Table 2). Membranes were examined in a G:Box (SynGene, UK) using the GeneSnap software package (Synoptics Ltd, SynGene). Analysis was done by ImageJ; Na_v_1.7 intensities were normalised to β-tubulin intensities in each of the 16 samples (samples from the ipsilateral and contralateral sides of 8 animals). Then, the ratio of the normalised intensities found on the ipsilateral and contralateral sides in each animal was calculated and averaged.

### Immunolabelling

The L4–L5 segments of the spinal cord and the L4 and L5 DRGs were dissected, post-fixed overnight in 4% paraformaldehyde and cryoprotected in 30% sucrose. Ten-micrometre sections were cut and incubated in PBS containing 0.3% Triton-X 100 (PBST) for 10 min, then in 10% normal donkey serum (NDS) for 1 h followed by the primary antibody (Supplementary Table 1). For visualisation of the phosphorylated serine 10 in histone H3 (p-S10H3) staining, the tyramide signal amplification procedure was used [12]. Other immunoreactions were visualised by Alexa Fluor-conjugated secondary antibodies (Supplementary Table 1). Slides were coverslipped with Vectashield (Vector Laboratories, UK) and examined with a Leica microscope attached to a Hamamatsu colour-chilled 3CCD camera.

### In vitro electrophysiology

The spinal cord slices were prepared using sham-injured and burn-injured Wistar rats as described previously [13]. One hour after the injury, 300-μm transverse slices were cut and incubated in dissection solution ((in mM) 95 NaCl, 1.8 KCl, 7 MgSO_4_, 0.5 CaCl_2_, 1.2 KH_2_PO_4_, 26 NaHCO_3_, 25 D-glucose and 50 sucrose) for 30 min at 35 °C, stored in a recording solution ((in mM) 127 NaCl, 1.8 KCl, 1.2 KH_2_PO_4_, 2.4 CaCl_2_, 1.3 MgSO_4_, 26 NaHCO_3_ and 25 D-glucose) at room temperature (21–24 °C) and allowed to recover for at least 1 h before recordings. All extracellular solutions were saturated with carbogen (95% O_2_, 5% CO_2_).

Whole-cell patch-clamp recordings (at 21–24 °C) were performed from the superficial dorsal horn neurons clamped at − 70 mV in the presence of 10 μM bicuculline and 5 μM strychnine in the bath solution as described previously [13]. The intracellular pipette solution contained (in mM) 125 gluconic acid lactone, 15 CsCl, 10 EGTA, 10 HEPES, 1 CaCl_2_, 2 Mg_2_ATP and 0.5 NaGTP and was adjusted to pH 7.2 with CsOH. An Axopatch 1D (Axon Instruments, USA) amplifier, a Digidata 1440A digitizer (Molecular Devices, USA) and the pCLAMP 10.5 software package were used for recordings. Low-pass filter (2 kHz), 10-kHz sampling rate and 80% series resistance compensation were used. Spontaneous excitatory post-synaptic currents (sEPSCs) with an amplitude of 5 pA or greater (at least twice of the noise) were included in the frequency and amplitude analyses. Basal activity recording was followed by recoding in the presence of ProTxII (10 nM in 0.1% BSA, Tocris) for 5 min. In the end of the recording protocol, capsaicin (200 nM) was applied to find whether the neuron received nociceptive input.

### Statistical analysis

Data were analysed as previously reported [12]. In brief, power calculations were used to estimate sufficient sample size using an online-based software (http://homepage.stat.uiowa.edu/~rlenth/Power/) and statistical analyses were carried out using the SPSS program (IBM SPSS statistics 22.0 for Windows). Data from immunostaining were analysed using a generalised linear model (GzLM), with significance assessed with the Wald chi-squared test. For Western blotting, independent *t* tests were used. In all cases, differences were regarded significant at *p* < 0.05. The statistical significance of ProTxII on sEPSCs was tested using paired *t* test and *t* test with Bonferroni correction for multiple comparisons. The sEPSC frequency after ProTxII application was also normalised against the pre-application control value. Data are expressed as mean ± standard error of mean; *n* refers to the number of biological repetitions.

## Results

### Antibody specificity

In our pilot experiments, we tested several anti-Na_v_1.7 antibodies and immunolabelling procedures on sections cut from rat L4 and L5 DRGs. While all the antibodies provided similar staining pattern, the antibody supplied by Millipore (Supplementary Table 1) and the procedure described above produced the highest signal-to-noise ratio, which we found suitable for quantitative analysis.

**Fig. 1:**
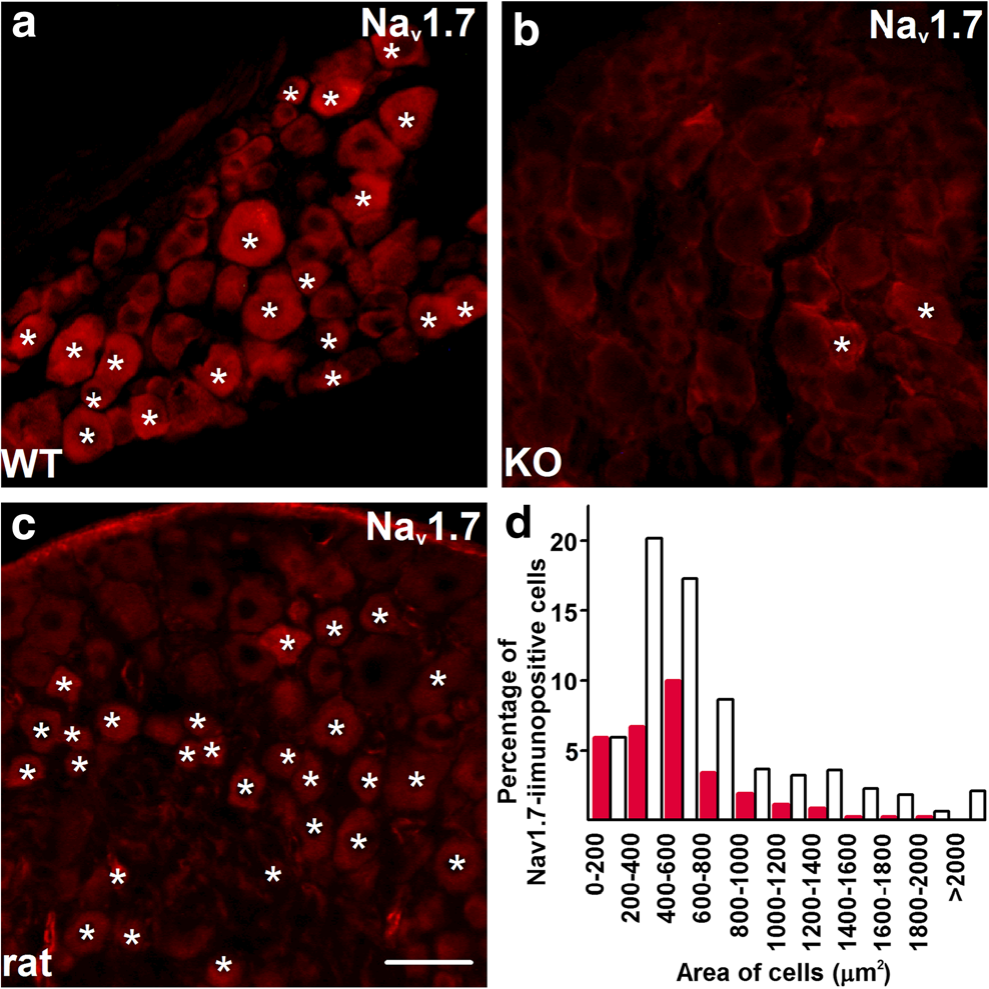
The anti-Na_v_1.7 antibody specifically identifies a sub-population of primary sensory neurons. **a** Incubation with the anti-Na_v_1.7 antibody (Millipore, AB5390) results in immunostaining in a group of primary sensory neurons in wild-type (WT) mice. Asterisks indicate immunopositive neurons. **b** The same anti-Na_v_1.7 antibody produced only faint staining in very few neurons in sections cut from the dorsal root ganglia dissected from mice lacking Na_v_1.7 (KO; asterisks). **c** The anti-Na_v_1.7 antibody also produces immunostaining in rat primary sensory neurons. Immunopositive cells are indicated by asterisks. **d** Cell size distribution of Na_v_1.7+ neurons in the L4 and L5 dorsal root ganglia of naive Sprague-Dawley rats. Note that most of the Na_v_1.7+ neurons are small- and middle-size cells. Empty bars indicate size distribution of all cells whereas red bars indicate the size distribution of neurons exhibiting Na_v_1.7 immunopositivity. Though we have not tested the expression of functional Na_v_1.7, the specificity and selectivity of the antibody suggest the expression of such functional channels. Scale bar = 50 μm on each microphotographs (Color figure online)

In WT mice, a significant proportion of neurons appeared immunopositive in the cytoplasmic compartment as well as in the cytoplasmic membrane (Fig. 1a). In contrast, only a few immunopositive neurons were apparent in sections cut from Na_v_1.7-cKO mouse DRG (Fig. 1b). The presence of a limited number of Nav1.7-immunopositive neurons was expected in Nav1.7-cKO mouse DRG, as neurons that do not express Nav1.8 may express Nav1.7 [14]. No immunostaining was observed in negative controls either when the primary antibody was replaced by normal serum on sections cut from WT mouse DRGs (data not shown) or when it was exhausted with the immunising peptide.

In rat L4–L5 DRGs, a significant proportion of neurons exhibited immunostaining (Fig. 1c). No immunopositive neurons were visible when the primary antibody was replaced by normal serum (data not shown) or exhausted with the immunising peptide (Supplementary Fig. 1a). In the positive control, an already characterised antibody (anti-TRPV1 antibody; [15]) showed the characteristic immunopositivity (Supplementary Fig. 1b).

Na_v_1.7 immunopositivity was found in about 25% of the neurons in naive rat DRGs (25.69 ± 6.03%, *n* = 4). The size distribution confirmed that Na_v_1.7 is expressed predominantly in small- and medium-size neurons (Fig. 1d). The average area of the Na_v_1.7-immunopositive neurons was significantly smaller than that of the Na_v_1.7-immunonegative cells (positive 406.14 ± 33.64 μm^2^, *n* = 4; negative 572.10 ± 33.33 μm^2^, *n* = 4; *p* = 0.0128, GzLM).

### Burn injury upregulates Na_v_1.7 expression in primary sensory neurons

**Fig. 2:**
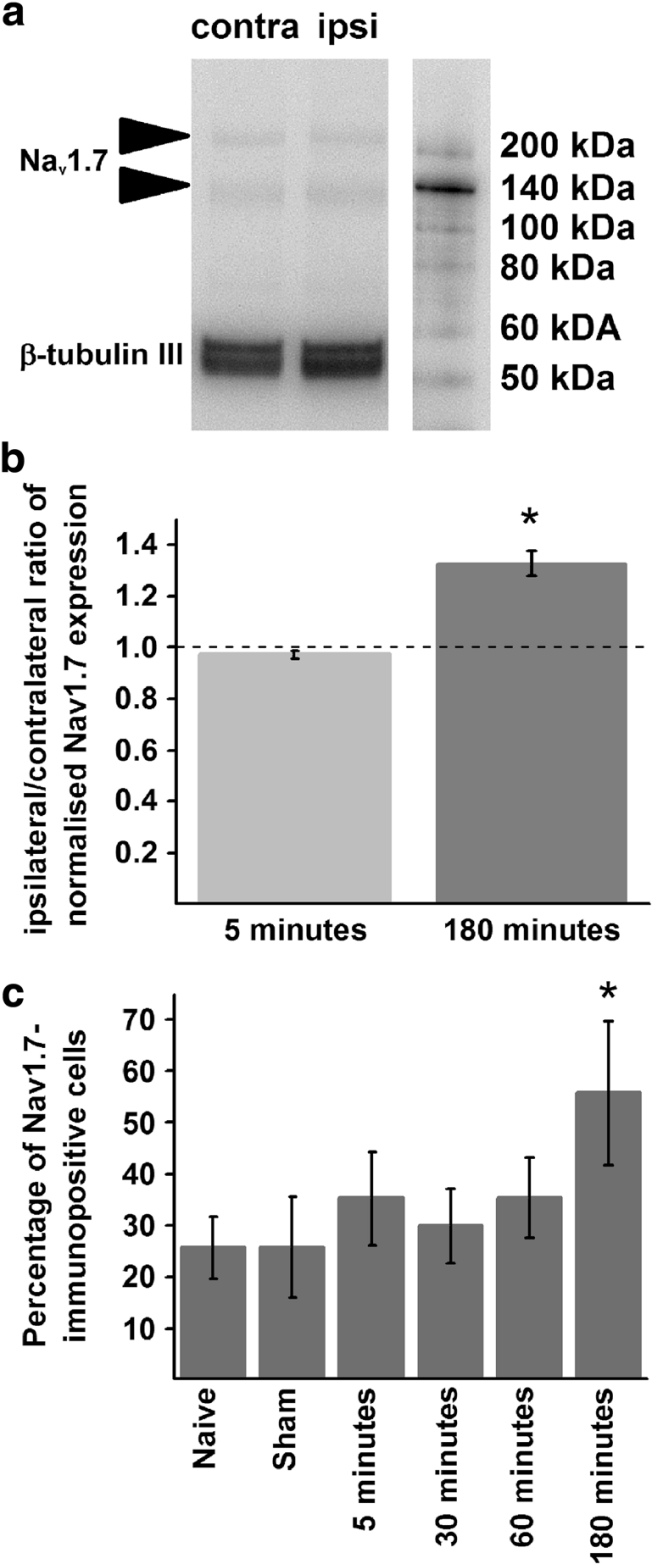
Burn injury induces upregulation in Na_v_1.7 expression. **a** A gel image of Western blotting using the anti-Na_v_1.7 and anti-β-tubulin III antibodies with protein samples isolated from the ipsilateral (ipsi) and contralateral (contra) L4 and L5 dorsal root ganglia of a rat 180 min after burn injury. Note that both antibodies produced double blots; consistent with previous findings, Na_v_1.7 is expressed at ~ 135 and 210 kDa (indicated by an arrowhead), whereas β-tubulin III expression is between 50 and 60 kDa. Both Na_v_1.7 bands were considered for analysis. **b** Ratios between normalised Na_v_1.7 expression found in the contra- and ipsilateral L4–L5 dorsal root ganglia 5 and 180 min after burn injury reveal that burn injury induces upregulation in Na_v_1.7 expression. *n* = 4. *p < 0.05. **c** Quantification of Na_v_1.7 immunostaining in L4–L5 dorsal root ganglia confirms upregulation of Na_v_1.7 expression at 180 min after burn injury. *n* = 4. *p < 0.01

Western blots of protein extracts from the ipsi- and contralateral DRGs of rats 5 min and 3 h after the induction of the burn injury showed the presence of two proteins corresponding to Na_v_1.7, at ~ 130 and ~ 210 kDa molecular masses (Fig. 2a) [16, 17]. Analysis of the Western blots revealed a significant increase in ipsilateral/contralateral ratio of Na_v_1.7 protein expression at 3 h post-injury (Fig. 2a, b), but not at the 5-min post-injury time point (Fig. 2b, Supplementary Fig. 2; 3 h 1.23 ± 0.09, *n* = 4; 5 min 0.94 ± 0.02, *n* = 4, *p* = 0.041, Student’s *t* test).

Quantification of Na_v_1.7-immunopositive neurons at various time points after the injury confirmed the upregulation of Na_v_1.7 expression in ipsilateral DRG at 3 h post-injury (naive 25.69 ± 6.03%, 3 h 55.81 ± 14.11%, *n* = 4 for each; *p* = 0.05, GzLM; Fig. 2c). However, no increase in the proportion of Na_v_1.7-immunopositive DRG neurons in contralateral DRGs at any time point following burn injury (not shown) or at either side following a sham injury was found (naive 25.08 ± 2.97%, *n* = 4, *p* = 0.693; sham ipsilateral 25.72 ± 9.81%, *n* = 4, *p* = 0.797; sham contralateral 27.67 ± 5.97%, *n* = 4, *p* = 0.857; 5 min 22.24 ± 2.57%, *n* = 4, *p* = 0.747; 30 min 40.05 ± 9.55%, *n* = 4, *p* = 0.185; 1 h 22.32 ± 8.28%, *n* = 4, *p* = 0.753; 3 h 18.96 ± 9.18%, *n* = 4, *p* = 0.532; GzLM).

### p-CREB is a marker for neuronal activation by burn injury

**Fig. 3:**
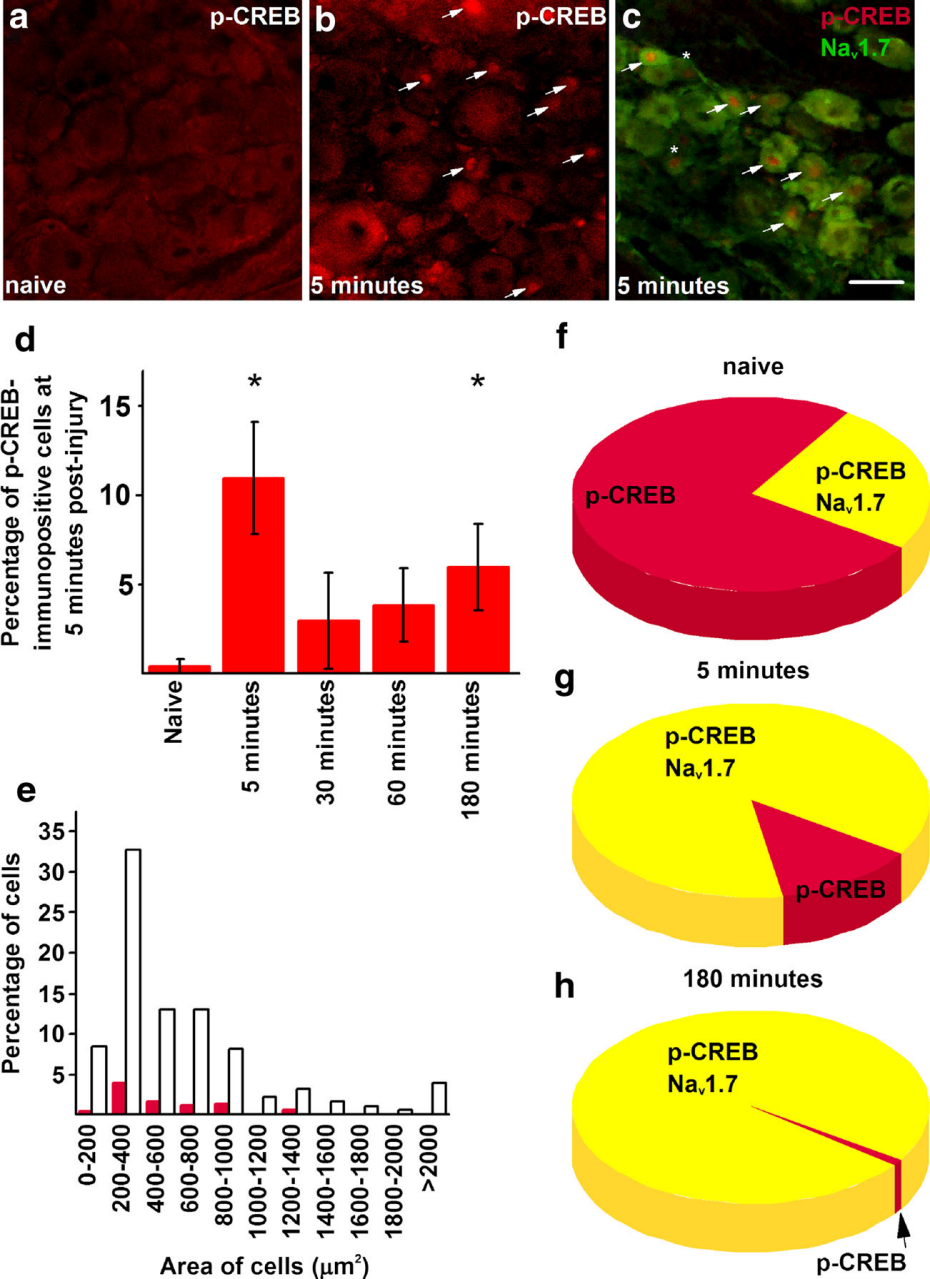
Burn injury induces upregulation in p-CREB expression in Na_v_1.7-expressing primary sensory neurons in L4–L5 dorsal root ganglia. **a** L4–L5 dorsal root ganglia dissected from naive rats contain virtually no neurons which express p-CREB in their nuclei. **b** Burn injury induces p-CREB expression in a significant number of primary sensory neurons in the ipsilateral L4–L5 dorsal root ganglia. Arrows indicate p-CREB-immunopositive nuclei. **c** Combined immunostaining with the anti-Na_v_1.7 (green) and the anti-p-CREB (red) antibodies on sections from the ipsilateral L4–L5 dorsal root ganglia shows that CREB phosphorylation following burn injury occurs predominantly in Na_v_1.7-expressing primary sensory neurons. Arrows show neurons exhibiting double immunopositivity, whereas asterisks indicate neurons with p-CREB immunopositivity without expressing Na_v_1.7. **d** Quantification of primary sensory neurons reveals significant upregulation of p-CREB expression at 5 and 180 min after burn injury in primary sensory neurons in the ipsilateral L4–L5 dorsal root ganglia. Asterisks indicate statistical significance. *n* = 4. **e** Size distribution of neurons exhibiting p-CREB expression in the nucleus (red bars) 5 min after the injury reveals that small-diameter neurons are activated by burn injury. Empty bars indicate all neurons. **f**–**h** Pie charts indicating the proportion of primary sensory neurons exhibiting Na_v_1.7 and p-CREB co-expression in the ipsilateral L4–L5 dorsal root ganglia in naive condition, 5 and 180 min after burn injury, respectively. Scale bar = 50 μm on each microphotograph (Color figure online)

Phosphorylated CREB (p-CREB) is a common downstream effector of various pathways implicated in regulating transcriptional changes associated with use-dependent increase in the activity and excitability (sensitisation) of primary sensory neurons by noxious stimuli [18]. Hence, we assessed whether p-CREB identifies activated primary sensory neurons in our burn injury model.

p-CREB expression exhibited a biphasic increase in primary sensory neurons following burn injury; at 5 min post-injury, p-CREB appeared in the nuclear compartment of small- and medium-size neurons (5 min 10.98 ± 3.16%, *n* = 4; naive 0.41 ± 0.41%, *n* = 4, *p* < 0.001; GzLM; Fig. 3a–d). Following a rapid return to baseline expression level (30 min 2.95 ± 2.68%, *n* = 4, *p* = 0.393; 60 min 3.85 ± 2.38%, *n* = 4, *p* = 0.287; GzLM), the proportion of p-CREB-expressing neurons was significantly increased again at 3 h post-injury (3 h 6.10 ± 2.41%, *n* = 4, *p* = 0.041 vs naive, *p* = 0.097 vs. 5 min, GzLM; Fig. 3d). The majority of neurons exhibiting immunopositivity for p-CREB were small- and medium-size neurons (Fig. 3e; positive 364.61 ± 50.78 μm^2^, *n* = 4; negative: 506.51 ± 26.69 μm^2^, *n* = 4; *p* = 0.001, GzLM). In addition to primary sensory neurons, p-CREB was also present in the nuclei of some satellite cells 5 min after the burn injury. However, further investigation of this expression was outside the scope of this study; therefore, we did not analyse satellite cell expression of p-CREB any further.

### p-CREB is expressed in Na_v_1.7-expressing neurons

Double immunostaining revealed that while in the naive rats, about 25% of the very few cells with p-CREB-immunopositive nuclei exhibited Na_v_1.7 immunopositivity, 5 min after the burn injury, ~ 90% of the cells with p-CREB-immunopositive nuclei also exhibited immunopositivity for Na_v_1.7 (87.5 ± 7.98%, *n* = 4; Fig. 3c, f, g). The co-expression pattern was very similar at 3 h post-injury (*p* = 0.983; Fig. 3h).

Very few Na_v_1.7-immunopositive neurons showed p-CREB immunopositivity in naive animals (13.3 ± 3.33%, *n* = 4). The co-expression pattern increased to around 80% 5 min after the burn injury (77.58 ± 10.16%, *n* = 4), and this proportion remained similar at 3 h post-injury (63.42 ± 10.31%, *n* = 4; *p* = 0.758).

Together, these data support recent reports on the pivotal role of Na_v_1.7 in the development of burn injury-associated pain [4]. Those reports also showed that Na_v_1.7 is particularly important in the development of burn injury-associated heat hyperalgesia [4]. We have shown most recently that burn injury induces a rapid and sustained upregulation of p-S10H3 in a sub-population of spinal dorsal horn neurons [12]. Our findings also indicate that p-S10H3 can be used as a marker for nociceptive activation of spinal cord neurons involved in the development of inflammatory heat hyperalgesia [12]. Therefore, next, we assessed the effect of blocking Na_v_1.7 on p-S10H3 expression in the spinal dorsal horn.

### Na_v_1.7 blockade partially reduces burn injury-induced nociceptive activation in spinal cord neurons

**Fig. 4:**
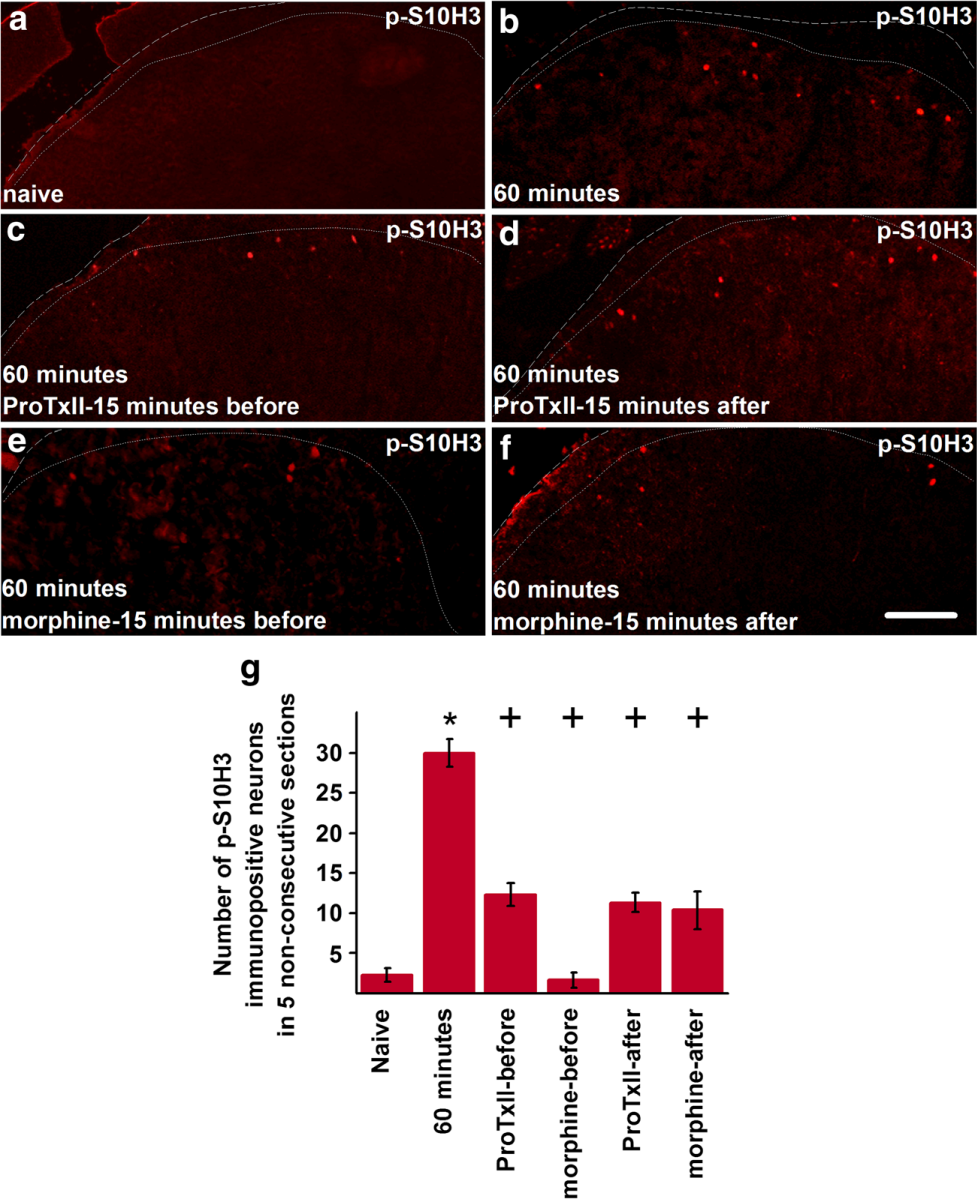
Intraperitoneal injection of protoxin II or morphine reduces burn injury-induced upregulation of p-S10H3 expression in the ipsilateral spinal dorsal horn. **a**–**f** p-S10H3 expression in the ipsilateral spinal dorsal horn in naive condition (**a**), 60 min after burn injury (**b**), with the injection of protoxin II 15 min before (**c**) or 15 min after (**d**) the injury and with the injection of morphine 15 min before (**e**) or 15 min after (**f**) the injury. Dashed lines indicate the border of the spinal cord, whereas dotted lines indicate the white-grey matter border. Note that burn injury induces upregulation in the expression of p-S10H3, whereas either protoxin II or morphine reduces that upregulation irrespective of whether the drug is given before or after the injury. **g** Quantification of neurons exhibiting p-S10H3 expression in various conditions. Asterisk indicates significant difference in the number of p-S10H3-expressing nuclei between naive condition and 60 min after burn injury, whereas plus signs indicate significant difference in the number of p-S10H3-expressing nuclei between 60 min after burn injury and various treatments. Scale bar = 100 μm on each image

Burn injury induced a significant increase in the number of neurons with nuclei immunopositive for p-S10H3 in the ipsilateral spinal dorsal horn at 60 min post-injury (60 min 30.00 ± 1.73, *n* = 3; control: 2.33 ± 0.88, *n* = 3; *p* < 0.001, GzLM; Fig. 4a, b, g). Neurons exhibiting nuclei with p-S10H3 immunopositivity were distributed among Na_v_1.7-immunopositive fibres (Supplementary Fig. 3). Intraperitoneal injection of ProTxII 15 min before the injury significantly reduced the number of pS10H3-positive nuclei in the ipsilateral spinal dorsal horn (Fig. 4a–c, g; ProTxII-before 12.33 ± 1.45, *n* = 3, *p* < 0.001, GzLM). ProTxII, injected 15 min after the injury, also significantly reduced the number of neurons exhibiting p-S10H3 expression in the ipsilateral spinal dorsal horn (Fig. 4a, b, d, g; ProTxII-after: 11.33 ± 1.20, *n* = 3, *p* < 0.001, GzLM). There was no difference between the reductions produced by protoxin injection before or after the injury (*p* = 0.491, GzLM; Fig. 4g).

Morphine injection 15 min before the injury completely prevented the upregulation of p-S10H3 expression by burn injury (morphine-before 1.66 ± 0.88, *n* = 3, *p* = 0.646, GzLM; Fig. 4a, b, e, g). Morphine, injected 15 min after the induction of the burn injury, significantly reduced the number of neurons exhibiting p-S10H3-immunopositive nuclei (morphine-after 11.33 ± 1.20, *n* = 3, *p* < 0.001, GzLM; Fig. 4a, b, f, g). The number of activated neurons found in the morphine-after group was not significantly different from that found in naive animals. Both ProTxII and morphine had similar effects on the expression of phosphorylated ERK (p-ERK) 1/2 in the spinal cord (Supplementary Fig. 4).

### ProTxII reduces sEPSC frequency following burn injury

**Fig. 5:**
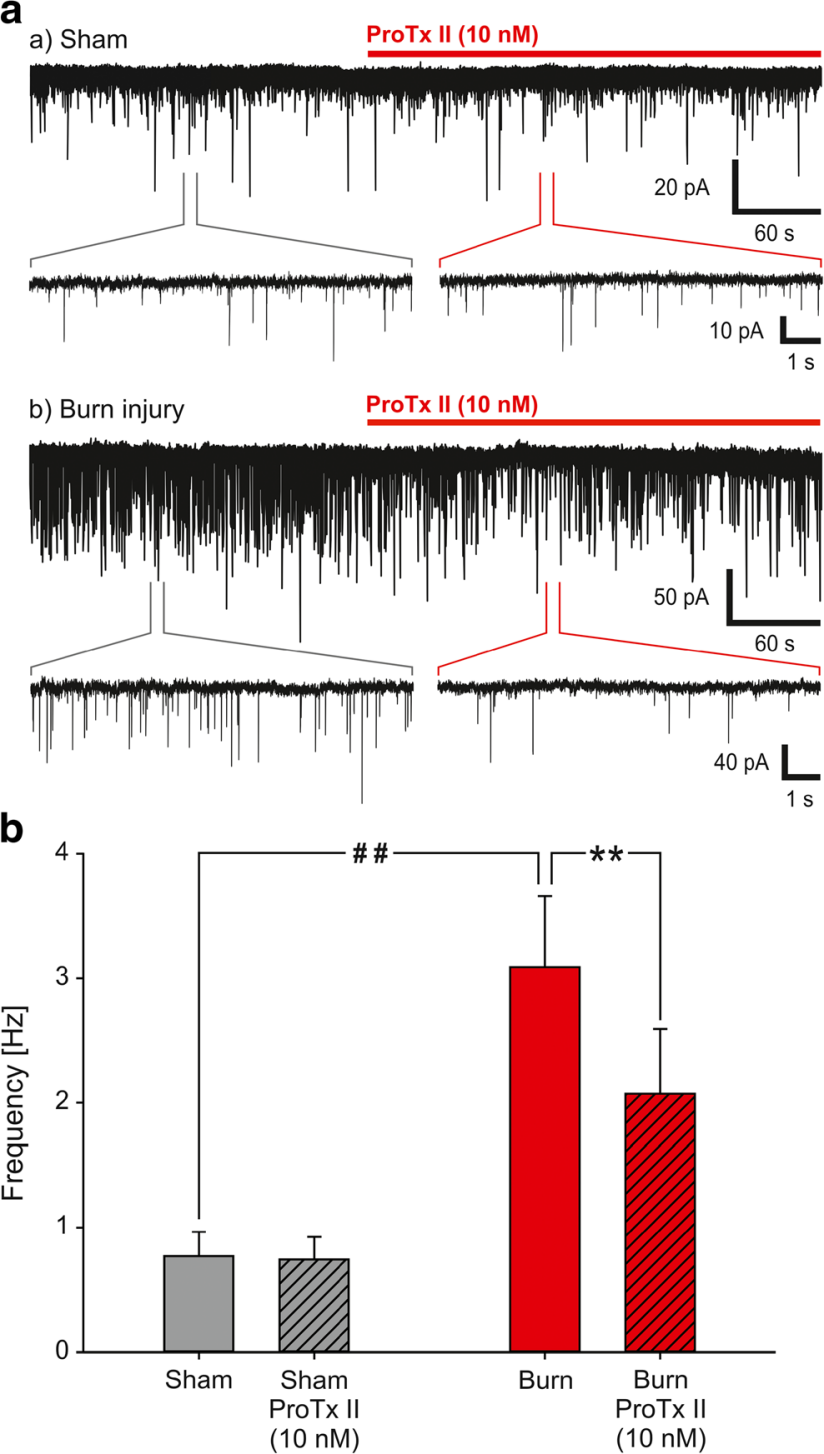
ProTxII significantly reduces sEPSC frequency following burn injury. The effect of ProTxII sEPSC frequency recorded from the spinal superficial dorsal horn neurons. In the slices with the sham treatment (*n* = 9), the basal sEPSC frequency was low and ProTxII (10 nM) did not produce any change (**a**, **b**, sham). Neurons in slices prepared after the burn injury (*n* = 10) exhibited a robust increase in sEPSCs (**a**, burn injury), and the application of ProTxII induced a significant decrease of the sEPSC frequency (**a**, **b**, **p = 0.006). The basal sEPSC frequency in the sham group and the burn injury group was significantly different (**b**, ##*p* = 0.002)

To confirm that ProTxII reduces spinal nociceptive processing in burn injury, we also assessed the effect of ProTxII on sEPSCs in the spinal superficial dorsal horn neurons. sEPSC frequency in sham-operated animals was 0.8 ± 0.2 Hz (*n* = 9), and ProTxII (10 nM) did not change that (99.7 ± 8.7% of the control value; Fig. 5a, b). sEPSC amplitudes in naive slices were − 14.7 ± 2.0 pA before and − 15.1 ± 2.2 pA after the ProTxII. All the tested neurons responded to capsaicin (200 nM; 25.6 ± 5.7 Hz, *n* = 8; *p* = 0.003).

sEPSC frequency exhibited a robust and significant increase following burn injury (3.1 ± 0.6 Hz; Fig. 5a, b, *p* = 0.002). ProTxII significantly decreased the sESPC frequency to 2.1 ± 0.5 Hz (66.2 ± 8.1% of the control value; Fig. 5a, b). The average sEPSC amplitude was − 17.8 ± 3.4 pA, and ProTxII did not change that (− 17.1 ± 3.6 pA). All neurons responded to capsaicin (31.5 ± 7.1 Hz; *n* = 10; *p* = 0.003). The capsaicin response was not different in the sham and injured groups.

## Discussion

Similar to previous reports, we found that a significant proportion of primary sensory neurons express Na_v_1.7 [5, 17]. While we did not test whether Na_v_1.7 is functional in the Na_v_1.7-expressing primary sensory neurons, previous findings that a significant proportion of primary sensory neurons do express such currents [4, 5, 14, 17, 19] indicate that at least a proportion of the Na_v_1.7-immunopositive neurons express functional Na_v_1.7 channels.

Both Western blotting and immunostaining revealed that Na_v_1.7 expression is increased in DRGs by 3 h post-injury. Similar upregulation has been reported in other peripheral inflammatory models [20, 21]. Increased density of Na_v_1.7-mediated currents in primary sensory neurons following burn injury was also reported recently [4]. The increased Na_v_1.7 expression and the increased density of Na_v_1.7-mediated currents, 3 h and 2 days after the injury, respectively, support the view that Na_v_1.7 significantly contributes in enhancing nociceptive signalling of primary sensory neurons during the entire course of burn injury [4].

Burn injury induced a biphasic upregulation in the expression of p-CREB, a marker for neurons activated by various painful peripheral pathologies, including inflammation of various origins in primary sensory neurons [18, 22]. The increase at 5 min could be due to the activation of neurons by the excessive heat and/or molecules released from the degenerated cells. The increase at 180 min could be due to the activation of neurons by inflammatory mediators [1]. Importantly, we found a high degree of co-expression between Na_v_1.7 and p-CREB after burn injury indicating that Na_v_1.7-expressing neurons are activated by this injury.

We analysed p-ERK 1/2 and p-S10H3 expressions to find the effect of morphine and ProTxII on spinal nociceptive processing. While p-ERK1/2 is well-established, p-S10H3 is a novel marker for nociceptive activation of spinal dorsal horn neurons [11, 12, 23]. As confirmed in the present study, burn injury induces sustained upregulation in both p-ERK1/2 and p-S10H3 expressions in the spinal dorsal horn [11, 12].

Both morphine and ProTxII, which respectively activates the μ-opioid receptors (MOR; [24]) and inhibits Na_v_1.7 [10], significantly reduced the burn injury-induced upregulation of both p-ERK1/2 and p-S10H3 expressions. While the finding that morphine reduces spinal nociceptive processing is in full agreement with a large body of previous findings [25, 26], the effect of ProTxII appears to be in contrast to previous reports that intravenous or intrathecal ProTxII injection does not reduce pain-related behaviour [10]. Although due to animal welfare considerations, we did not assess pain-related behaviour, the similar magnitude of inhibitory effects by morphine and ProTxII administration following the injury suggests that similar to morphine [27], ProTxII is also highly likely to produce an analgesic effect.

The lack of effect by ProTxII on pain-related behaviour was attributed to the inability of the toxin to access Na_v_1.7 in intact peripheral nerves and to pass the blood-brain barrier [10, 28]. However, a recent finding has demonstrated that ProTxII can access Na_v_1.7 in the spinal cord following intrathecal delivery as well as the peripheral nerve after perineural application [29]. Our data suggest that in addition to those, ProTxII may also reach Na_v_1.7 following intraperitoneal injection. While we did not assess the site of action, based on previous findings, we propose that the ProTxII-produced inhibitory effect on spinal nociceptive processing could be due to ProTxII-induced inhibition of Na_v_1.7 expressed on free nerve endings at the injured tissues as well as the central terminals of Nav1.7-expressing primary sensory neurons.

The significantly larger effect of morphine than of ProTxII on the upregulation of both p-S10H3 and p-ERK1/2 when applied before the injury could be due to the differing respective access to MOR and Na_v_1.7 of morphine and ProTxII in various parts of primary sensory neurons in naive conditions [25, 26].

The effect of morphine or ProTxII applied 15 min after the injury that models the time course of burn-injured patients receiving analgesics for the first time shows that both drugs are able to induce a significant downregulation in the expression of both markers, hence reducing spinal nociceptive processing. Interestingly, ProTxII produces a greater downregulation in p-S10H3 than p-ERK1/2 expression, which could be due to both Na_v_1.7 and p-S10H3 being involved in the development of heat hypersensitivity [4, 7, 8, 12], whereas p-ERK1/2 is involved in the development of both thermal and mechanical hypersensitivities [23]. Nevertheless, the downregulation of p-ERK1/2 and p-S10H3 by morphine or ProTxII 15 min after the injury indicates that ongoing activity of MOR- and/or Na_v_1.7-expressing primary sensory neurons is needed for the nociceptive activation of the spinal dorsal horn neurons in burn injury.

Our electrophysiological recordings confirm the significant role of Na_v_1.7 in spinal nociceptive processing following burn injury. Burn injury significantly increased sEPSC frequency which was significantly reduced by ProTxII. The differential effect of ProTxII in naive slices and slices prepared after burn injury could be due to Na_v_1.7 playing a minor role, whereas it gains a much more prominent role in generating spontaneous activity of nociceptive primary sensory neurons in naive condition and after burn injury, respectively. Alternatively, ProTxII, due to neuroinflammatory processes in the spinal cord, may have access to Na_v_1.7 on the central terminals of the primary sensory neuron after burn injury.

Morphine or other opioids used currently to control pain in burn-injured patients induce a series of undesirable effects [30, 31]. Based on the similar effects of morphine and ProTxII on p-ERK1/2 and p-S10H3 expressions by the spinal dorsal horn neurons and the role of p-ERK1/2 and p-S10H3 in the development of persistent pain associated with peripheral pathologies [11, 12, 32, 33], we propose that blocking Na_v_1.7 could reduce pain, particularly heat hyperalgesia [4, 7, 8], in burn injury with a potency equivalent to that produced by morphine.

Previous attempts to recapitulate the profound analgesic phenotype of Na_v_1.7^−/−^ mice or loss of function human mutations [5, 19] by pharmacological agents produced disappointing results [3]. However, a recent report has shown that a ProTxII-based designer peptide acting on Na_v_1.7 is able to reproduce the analgesic phenotype observed in mice lacking Na_v_1.7^−/−^ or humans having a loss of function Na_v_1.7 mutations [29]. Therefore, based on the expression pattern of Na_v_1.7 [5, 14, 17, 19], it is likely that blocking Na_v_1.7, particularly in a cell-specific manner, could produce a significant analgesic effect with significantly less undesirable effects than opioids.

## Electronic supplementary material

ESM 1 (PDF 546 kb).
