# Supplementary material for: The NAv1.7 blocker protoxin II reduces burn injury-induced spinal nociceptive processing
Source: J Mol Med (Berl). 2017 Oct 23;96(1):75–84. doi: 10.1007/s00109-017-1599-0 (PMC5750333; doi:10.1007/s00109-017-1599-0)
Supplement: Supplementary file 1 — (PDF 546 kb). [file 109_2017_1599_MOESM1_ESM.pdf]

Electronic supplementary material (ESM)

J Mol Med 2017

The $Na_v1.7$ blocker protoxin II reduces burn injury-induced spinal nociceptive processing

Jose Vicente Torres-Pérez[1], Pavel Adamek[2,3], Jiri Palecek[2], Marcela Vizcaychipi[4], Istvan Nagy[1], Angelika Varga[5]

[1]Nociception Group, Section of Anaesthetics, Pain Medicine and Intensive Care, Department of Surgery and Cancer, Imperial College London, Chelsea and Westminster Hospital, 369 Fulham Road, London, SW10 9NH, UK; [2]Department of Functional Morphology, Institute of Physiology of the Czech Academy of Sciences, Czech Republic; [3]Department of Physiology, Faculty of Science, Charles University, Czech Republic; [4]Department of Anaesthetics, Chelsea and Westminster Hospital NHS Trust, 369 Fulham Road, London, SW10 9NH, UK; [5]MTA-DE-NAP B-Pain Control Research Group, Department of Anatomy, Histology and Embryology, University of Debrecen, Debrecen, H-4012

Running title: $Na_v1.7$ and burn injury

Corresponding author
Istvan Nagy
Nociception Group,
Section of Anaesthetics, Pain Medicine and Intensive Care,
Department of Surgery and Cancer,
Imperial College London,
Chelsea and Westminster Hospital,
369 Fulham Road,
London,
SW10 9NH,
UK
Phone: 44-(0)20-8746-8897
Fax: 44-(0)20-3315-5119
Email: i.nagy@imperial.ac.uk

Supplementary materials

Table S1. List of primary antibodies used and corresponding conditions of usage. IHC: immunohistochemistry; WB: western blotting; IHC-TSA: IHC combined with tyramide signal amplification.

Primary antibody	Supplier	Catalogue	Host	Application
β-tubulin III	Sigma	T8578	mouse	1:7,000 IHC, 1:1,000 WB
Na_v1.7	Millipore	ab5390	rabbit	1:750 IHC, 1:2,000 WB
NeuN	Millipore	mab377	mouse	1:2,000 IHC
pCREB	Cell Signalling	9198	rabbit	1:5,000 IHC
pERK1/2	Neuromics	RA15002	rabbit	1:1,500 IHC
pS10H3	Santa Cruz	sc8656R	rabbit	1:750 IHC-TSA
TRPV1	Neuromics	GP141000	guinea pig	1:1,500 IHC

Supplementary figures

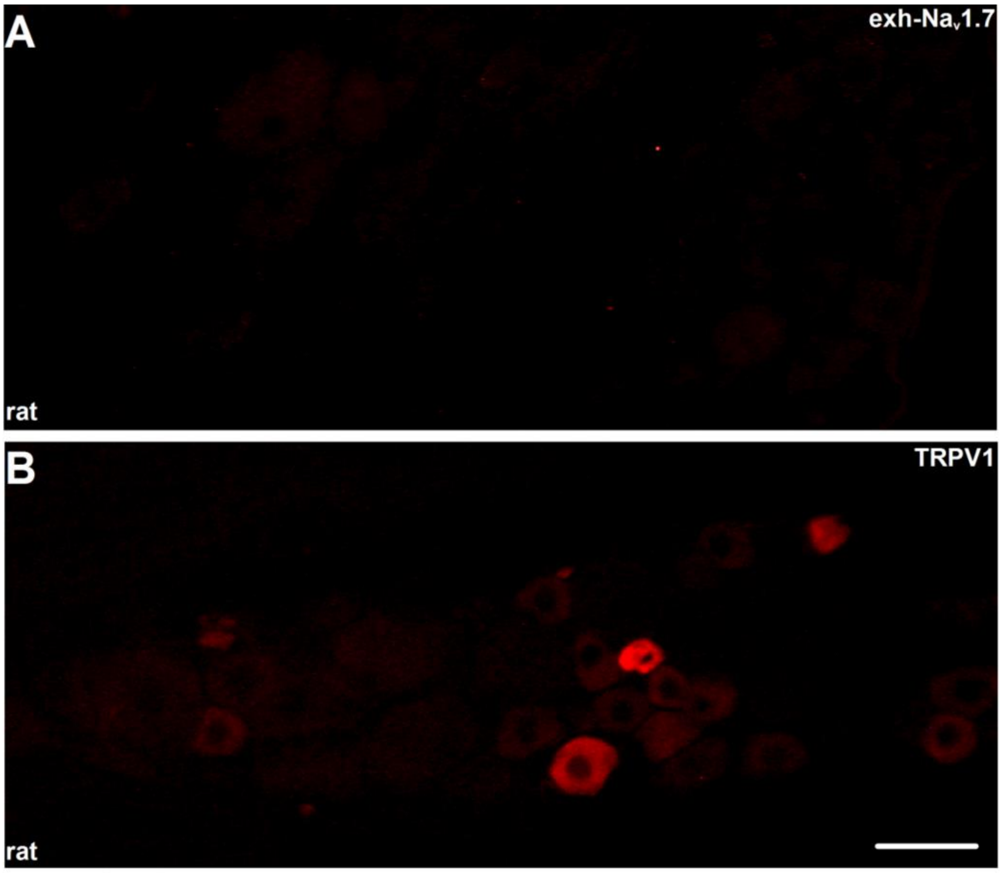

Figure S1.

Controls for immunostaining with the anti-Na$_v$1.7 antibody on L4-L5 dorsal root ganglia dissected from naive rats. (A) shows lack of immunoreaction following the exhaustion of the primary antibody. (B) shows a positive control when a well characterised anti-TRPV1 antibody was used as the primary antibody. Scale bar=50µm.

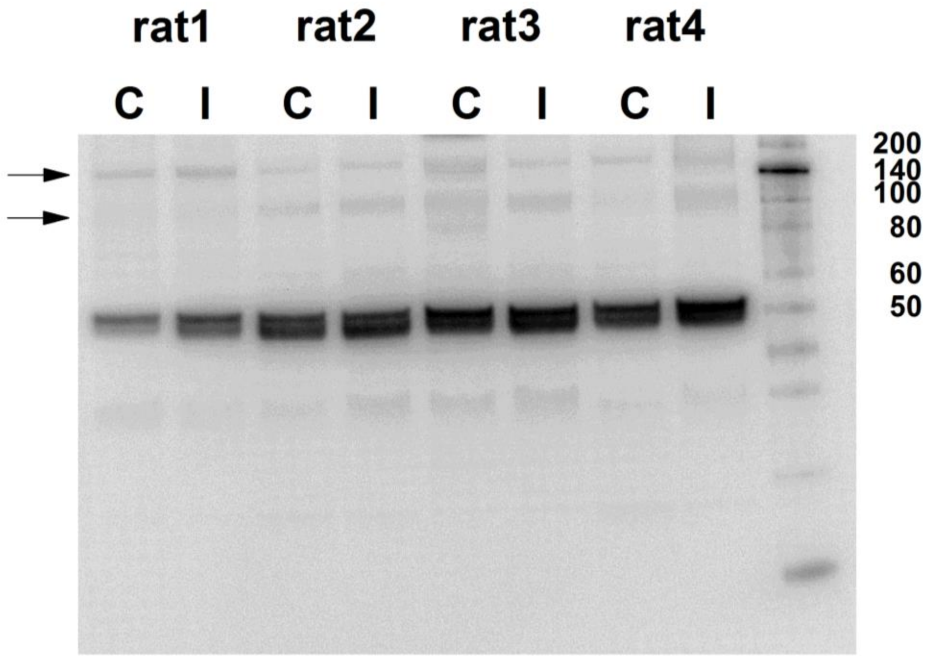

Figure S2.

A gel image of Western blotting using the anti-Na_v1.7 and an anti-β-tubulin III antibody with protein samples isolated from the ipsilateral (ipsi) and contralateral (contra) L4 and L5 dorsal root ganglia of a rat 5 minutes after burn injury. Note that both antibodies produced double bands; consistently with previous findings, Na_v1.7 is expressed at ~135 and 210kD (indicated by arrowhead), whereas β-tubulin III expression is between 50 and 60 kD. Both Na_v1.7 bands were considered for analysis.

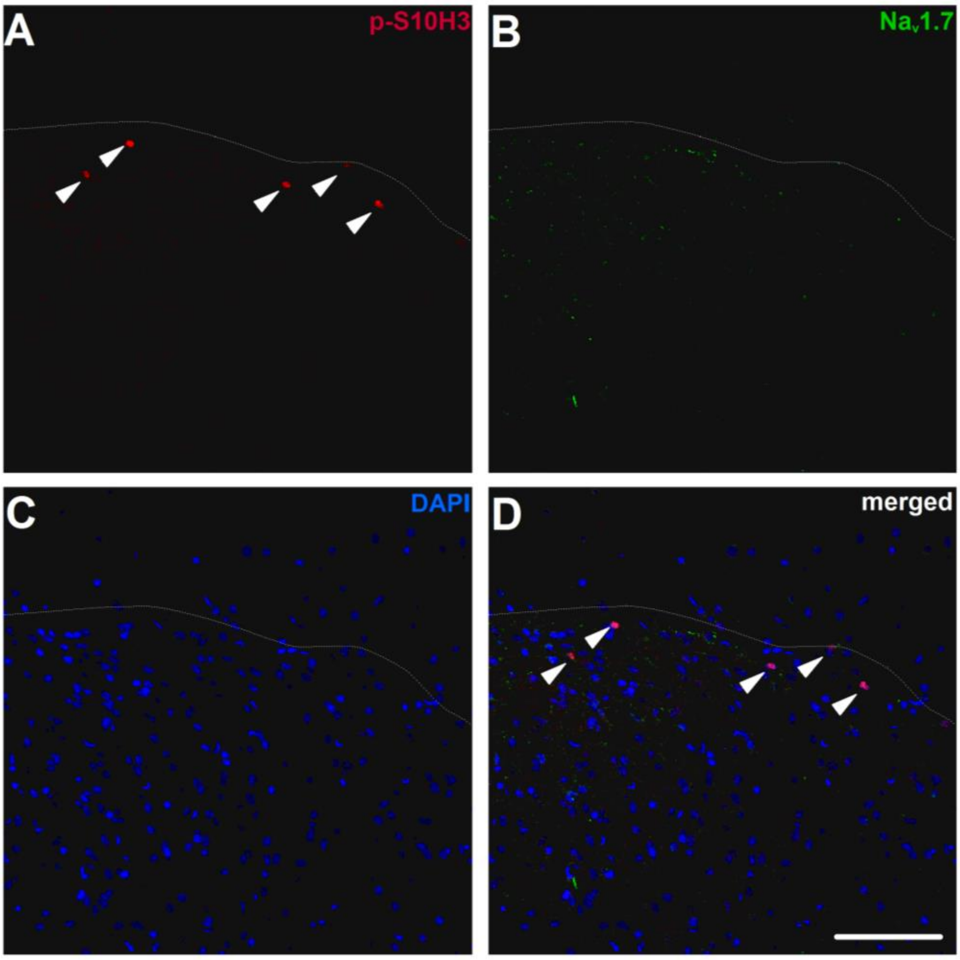

Figure S3.

Combined immunostaining with the anti-Na$_v$1.7 (green) and an anti-p-S10H3 (red) antibody of a section cut from the ipsilateral spinal dorsal horn of a rat 60 minutes after burn injury. The section is also stained with DAPI to show nuclei. Arrow heads indicate p-S10H3-expressing nuclei. The dotted line indicates the border between the white and grey matter.

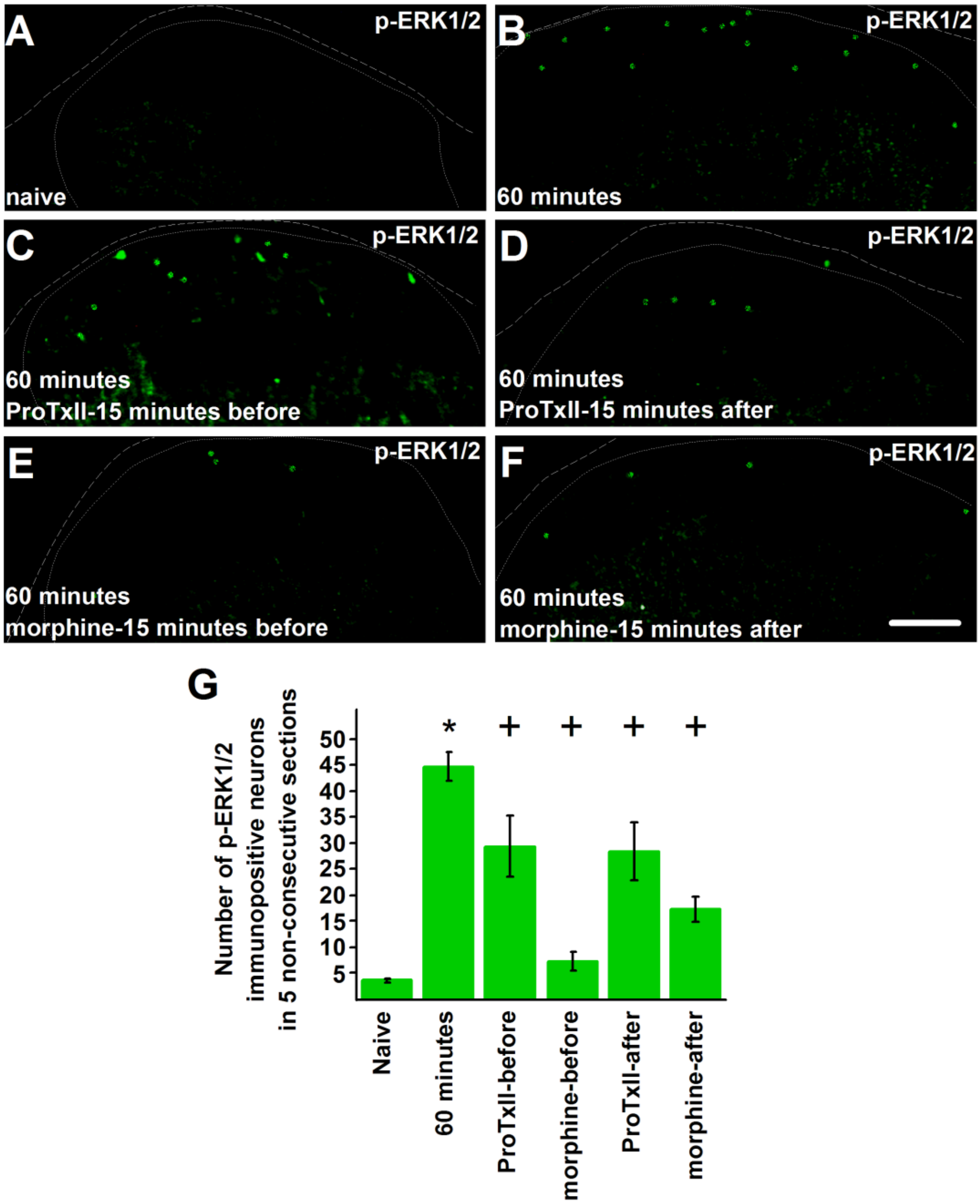

Figure S4.

(A-F) p-ERK1/2 expression in the ipsilateral spinal dorsal horn in naive condition (A), 60 minutes after burn injury (B), with the injection of protoxin II 15 minutes before (C) or 15 minutes after (D) the injury and with the injection of morphine 15 minutes before (E) or 15 minutes after (F) the injury. Note that burn injury induces

up-regulation in the expression of p-ERK1/2, whereas both protoxin II and morphine reduce that up-regulation irrespective whether the drug is given before or after the injury.

(G) Quantification of neurons exhibiting p-ERK1/2 expression in various conditions. Asterisk indicates significant difference in the number of p-ERK1/2 expressing neurons between naive and 60 minutes after burn injury, whereas plus signs indicate significant difference in the number of p-S10H3 expressing neurons between 60 minutes after burn injury and various treatments.

Scale bar=100μm on each image.
